# Artificial Neural Network Individualised Prediction of Time to Colorectal Cancer Surgery

**DOI:** 10.1155/2019/1285931

**Published:** 2019-07-09

**Authors:** N. J. Curtis, G. Dennison, E. Salib, D. A. Hashimoto, N. K. Francis

**Affiliations:** ^1^Department of Surgery and Cancer, Imperial College London, Level 10, St. Mary's Hospital, Praed Street, London W2 1NY, UK; ^2^Department of General Surgery, Yeovil District Hospital NHS Foundation Trust, Higher Kingston, Yeovil BA21 4AT, UK; ^3^Faculty of Health and Life Sciences, University of Liverpool, Brownlow Hill, Liverpool L69 7ZX, UK; ^4^Department of Surgery, Massachusetts General Hospital, 55 Fruit Street, Boston, MA 02114, USA; ^5^Faculty of Science, University of Bath, Wessex House 3.22, Bath BA2 7AY, UK

## Abstract

**Aim:**

Colorectal cancer pathway targets mandate prompt treatment although practicalities may mean patients wait for surgery. This variable period could be utilised for patient optimisation; however, there is currently no reliable predictive system for time to surgery. If individualised surgical waits were prospectively known, tailored prehabilitation could be introduced.

**Methods:**

A dedicated, prospectively populated elective laparoscopic surgery for colorectal cancer with a curative intent database was utilised. Primary endpoint was the prediction of the individualised waiting time for surgery. A multilayered perceptron artificial neural network (ANN) model was trained and tested alongside uni- and multivariate analyses.

**Results:**

668 consecutive patients were included. 8.5% underwent neoadjuvant chemoradiotherapy. The mean time from diagnosis to surgery was 53 days (95% CI 48.3-57.8). ANN correctly identified those having surgery in <8 (97.7% and 98.8%) and <12 weeks (97.1% and 98.8%) of the training and testing cohorts with area under the receiver operating curves of 0.793 and 0.865, respectively. After neoadjuvant treatment, an ASA physical status score was the most important potentially modifiable risk factor for prolonged waits (normalised importance 64%, OR 4.9, 95% CI 1.5-16). The ANN findings were accurately cross-validated with a logistic regression model.

**Conclusion:**

Artificial neural networks using demographic and diagnostic data successfully predict individual time to colorectal cancer surgery. This could assist the personalisation of preoperative care including the incorporation of prehabilitation interventions.

## 1. Introduction

Despite advances in surgical and perioperative colorectal care, many patients still develop early comorbidity which risks poor clinical, functional, and long-term outcomes [1–3]. The concept of prehabilitation, where patients undertake risk factor assessments to determine their baseline and identify impairments allowing targeted preoperative multimodality interventions, is aimed at reducing perioperative morbidity [4]. Early data suggests multimodal prehabilitation programmes can improve postoperative pain, length of stay, early morbidity, and physical function following major abdominal surgery including colorectal cancer resection [5, 6].

Prehabilitation represents a logical evolution of perioperative care, but its wide application seems to be presently limited by logistics and organisational barriers. Regulatory guidelines requiring prompt cancer treatment can conflict with optimum implementation of prehabilitation programmes. Whilst data from a number of randomised trials is awaited, currently, there is insufficient evidence to support the alteration of preoperative patient care pathways [7, 8].

Our research group along with others has reported that time from colorectal cancer diagnosis to curative laparoscopic surgery varies [9, 10]. Although the average time was 53 days and time from primary care presentation was 17 weeks, no detrimental impact on overall survival was seen [9, 10]. This window could therefore be potentially used for prehabilitation and patient optimisation with the goal of reducing morbidity.

In order to develop a personalised prehabilitation programme, prospective individualised prediction of likely waiting time is first required. Modern intelligent data analysis tools such as machine learning offer new opportunities for predictive tool development [11, 12]. Artificial neural networks (ANN), inspired by biological nervous systems, process data in computational units weighted by the previous experience of outcome data [11, 13]. These systems learn and improve with each use and would incorporate ongoing developments in perioperative practices. ANN have been successfully applied to a number of surgical fields with some improvements over traditional analyses reported [12, 14–16].

To allow prehabilitation planning on an individualised patient basis, a reliable prediction model allowing early identification of expected surgical waiting times is required, but currently, there is no method for this. Therefore, we aimed to investigate if artificial neural networks using patient and tumour factors could reliably predict time from diagnosis to curative laparoscopic colorectal surgery.

## 2. Methods

A dedicated previously reported colorectal cancer patient database was reviewed [9]. The inclusion criteria were patients receiving laparoscopic surgery with curative intent for colorectal cancer between 2002 and 2015. Distant metastatic disease, nonelective status, and open surgical approaches were excluded [9]. Diagnosis date (colorectal cancer multidisciplinary meeting date where cancer findings and treatment plan were agreed), patient demographics, and tumour characteristics were captured [9]. As previously reported, no operations were purposely delayed, and we were not using any formalised prehabilitation care pathway during these years [9]. The operation date was the earliest possible that was acceptable to the patient after all required investigations and preoperative preparations were performed with the availability of a specialist theatre and surgical team [9].

As previously described, patients were dichotomised using four-, eight-, and twelve-week waiting times as these represented clinically relevant timepoints as well as our institution's mean wait and boundaries of the 95% confidence interval [9]. The primary outcome of this study was the accuracy of ANN waiting time prediction for each timepoint.

### 2.1. Artificial Neural Network

ANN is a computational model composed of a large number of highly interconnected processing elements (neurons) working in unison. Neural networks process information in a similar way the human brain does, and like people, ANN learn by example. Detailed ANN descriptions have previously been provided [11, 13, 16]. Our ANN was created with factors that would be routinely available at the time of diagnosis: gender, age, American Society of Anaesthesiologists (ASA) score, body mass index, tumour location, staging details, and treatment plan (neoadjuvant treatment and/or need for an ostomy). An input layer, one hidden layer, and an output layer design were adopted. 70% of the cohort was randomly selected for ANN training which was then tested on the remaining patients.

The relative weight of each variable was explored and reported as normalised importance where the most important variable in each ANN analysis was scored as 100%.

### 2.2. Artificial Neural Network Cross-Validity

Receiver operator characteristics curves (ROC), area under the curve (AUC), gain and lift charts, and comparison with logistic regression modelling were used for the cross-validation of the ANN. The predictive quality of our ANN was tested using the data of patients in the dataset that had not been used in the training phase.

### 2.3. Univariate and Multivariate Analyses

Data was managed with SPSS (version 24.0; IBM, NY, USA). Categorical variables were analysed with cross tabulation and chi-squared or Fisher's exact test with calculations of odds ratios to assess associations between groups. Multivariate association between variables was assessed using binary logistic regression with only those variables identified as being significantly associated with diagnosis to treatment in the univariate analyses which were included in the modelling process. The data was screened for potentially influential observations, and the extent of multicollinearity amongst predictor variables was examined using variance inflation factors. The sample size is sufficiently large to ensure stable logistic regression parameter estimates obtained which are not suspect on accuracy or precision. The odds ratio for individual variables is computed from the regression equation as OR = *e* ^*B*^, adjusted for all other variables simultaneously. The effect magnitude was quantified using the odds ratio (OR) with 95% confidence interval. Unless stated otherwise, results are reported as the median (interquartile range).

## 3. Results

668 consecutive patients met the inclusion criteria and underwent laparoscopic resection with curative intent. 35 patients were excluded from the ANNs due to incomplete data. Cohort data are displayed in Table 1. The mean time from diagnosis to surgery has been previously reported as 53 days [11]. 570 (85%) patients had surgery within 12 weeks of diagnosis. 441 (70%) were selected as training cases with 192 (30%) used to test the predictive function of the ANN.

### 3.1. Four Weeks

ANN analysis for correct prediction of time to surgery above or below four weeks was 62%. The model was better at identifying those waiting longer than four weeks (70.6% and 72% of the training and testing cohorts, respectively) than under 4 weeks (51.8% and 49.4%). The area under the curve was 0.641 with gain and lift charts (supplementary figure 1a-c) suggesting a lack of clinical utility for ANN prediction for the four-week timepoint. Modifiable risk factors (BMI and ASA) were seen to be the most important influence on time to surgery (normalised importance 100% and 67.5%) with tumour stage and location also identified as key variables (69.4% and 63%, Table 2).

### 3.2. Eight Weeks

**Figure 1:**
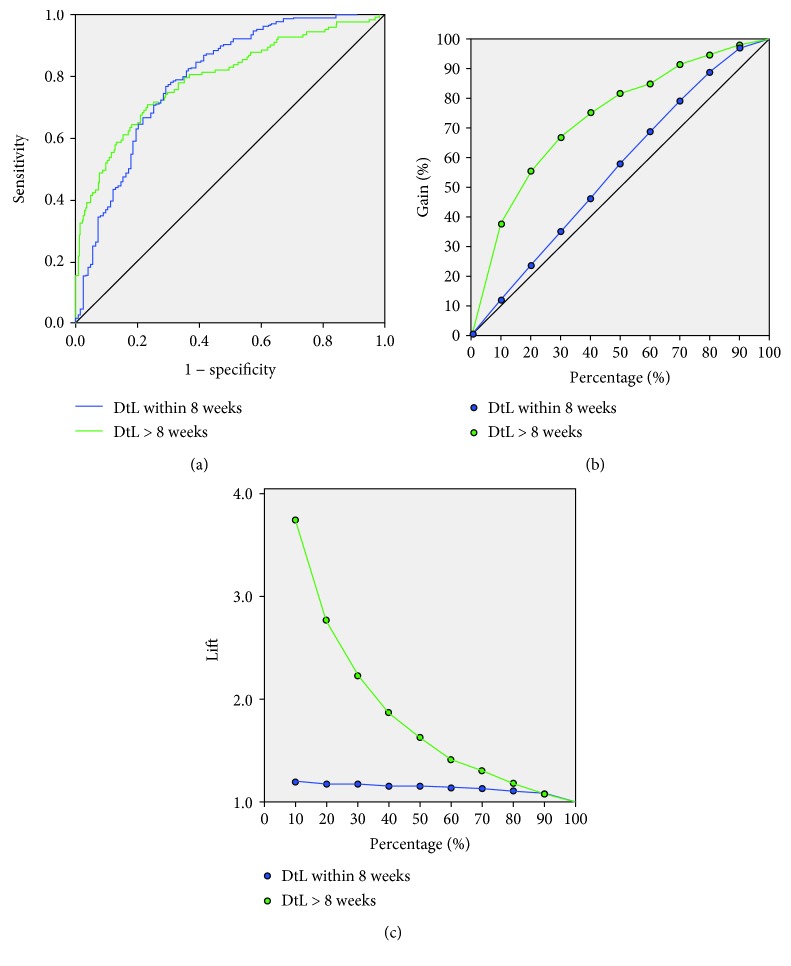
(a) ROC for the eight-week analysis. AUC for prediction of time to surgery was 0.793. (b) Gain chart shows ANN utility of predicting times longer than eight weeks. (c) Lift chart. Using 10% of the cohort, the ANN was 3.8 times more likely to correctly predict time to surgery over random selection. DtL: diagnosis to laparoscopy.

**Figure 2:**
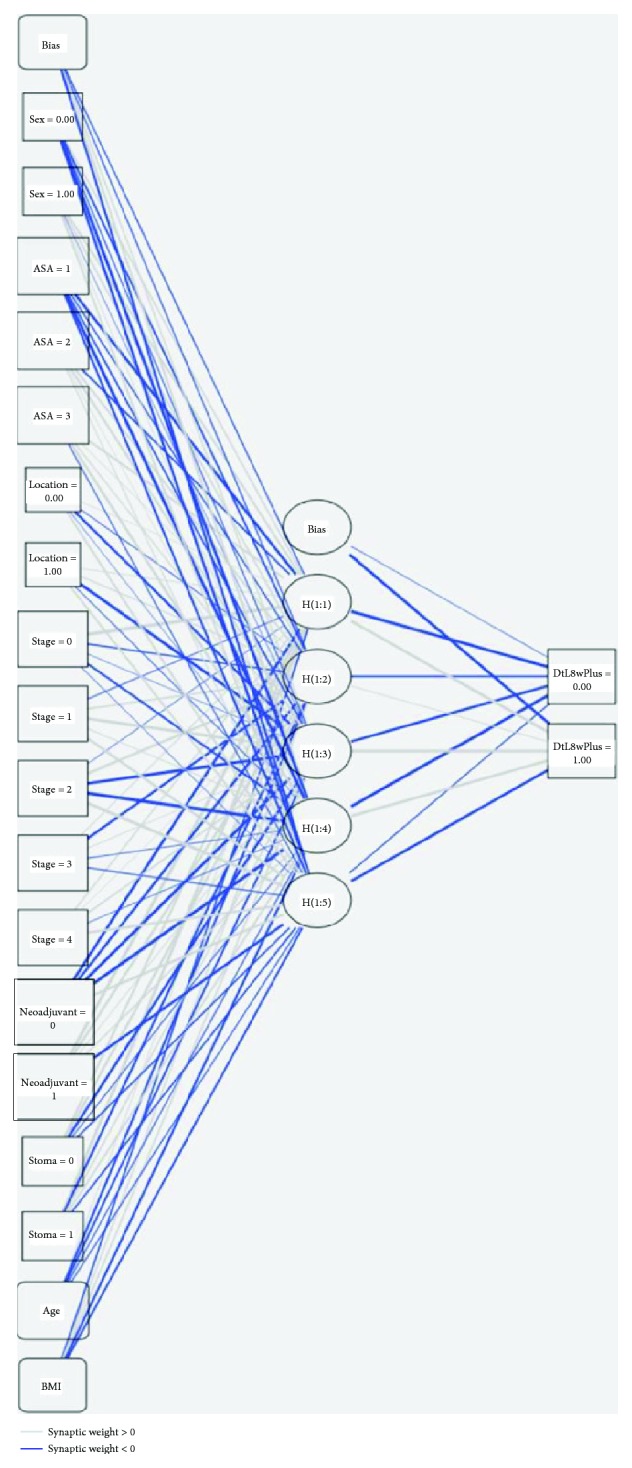
The neuronal links and strengths for the eight-week ANN. Graphical representation facilitates investigation of the identified associations and their strengths. ANN is inspired by the way the human brain processes information. It is composed of a large number of highly interconnected processing elements (neurons) working in unison to solve specific problems. Our example is the commonest type of artificial neural network consisting of three layers: “inputs” connected to “hidden” units, which are connected to a layer of “output” units. The activity of the input units represents the raw information that is fed into the network. The inputs are “weighted,” with the effect that each input has at decision making which is dependent on the weight of that particular input. These weighted inputs are then added together through an adder function (linear combiner) for computing the weighted sum of the inputs. The behaviour of each hidden unit is determined by the activities of the input units and the weights on the connections between the input and the hidden units. Output units depend on the activity of the hidden units and the weights between the hidden and output units. If they exceed a preset threshold value, the neuron fires. In any other case, the neuron does not fire. This ANN could identify targets for quality improvement efforts to improve clinical practices. Abbreviations: DtL: diagnosis to laparoscopy; DtL8weeksPlus: patients waiting under (0) or over [1] eight weeks from diagnosis to laparoscopy; ASA: American Society of Anaesthesiologists score; BMI: body mass index; H: “Hidden” unit layer. In this figure, 0 reflects no/not used/female gender/colonic cancer as appropriate with 1 denoting yes/positive/males/rectal cancer as appropriate.

The ANN correctly predicted those going to surgery inside eight weeks in 97.7% and 98.8% of the testing and training cohorts, respectively, with an AUC of 0.793 (Figure 1(a)). The neuronal links are displayed in Figure 2. The ANN could not accurately identify those waiting longer than eight weeks (37.5%) although the lift chart demonstrates 3.8 times more long waiting patients than randomly selecting 10% of the patients without the model. Gain charting showed the top 70% of the sample identified 90% of patients waiting over eight weeks (Figures 1(b)–1(c)).

### 3.3. Twelve Weeks

**Figure 3:**
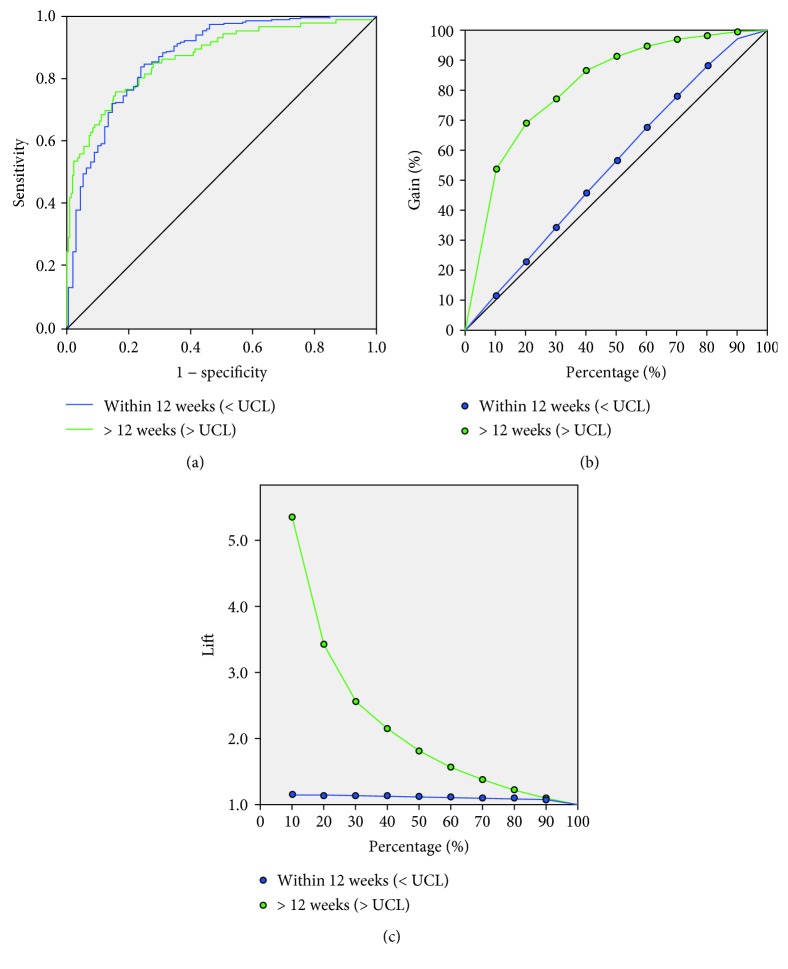
ROC (a), gain (b), and lift (c) charts for the 12-week ANN analysis. Stronger results are seen with an AUC of 0.865 and a lift of 5.2 using 10% of randomly selected patients. DtL: diagnosis to laparoscopy.

For the twelve-week assessment, the ANN correctly identified 97.1% and 98.8% of the training and testing groups, respectively, with an overall accuracy of 90.9% (Table 3). ROC analysis showed an AUC of 0.865 for prediction (Figure 3(a)). Gain charting and lift data showed the ANN was up to five times more likely to correctly identify long waiters and the top 50% of the sample identified 90% of the long waiters (Figures 3(b) and 3(c)). The 12-week neuronal links are displayed in supplementary figure 2.

The importance of individual factors is shown in Table 2. For the eight- and twelve-week analyses, undergoing neoadjuvant treatment was the strongest factor for longer waits followed by patient age. ASA scores were the only potentially modifiable risk factor identified in this analysis and was more important for the eight-week timepoint (63.7% and 48.1%, respectively).

### 3.4. Artificial Neural Network Cross-Validity

Based on the ANN results, cross-validity was explored using a logistic regression analysis of the eight-week data. ANN results were concordant with data from uni- (Table 1) and multivariate analyses (Table 4). Neoadjuvant therapy represented the highest risk of delayed surgery (OR 16.8, 95% CI 8.2-34.4) with ASA representing the only potentially modifiable factor (OR 1.6, 95% CI 1.5-16). A binary logistic regression model prediction score was calculated from all factors in the multivariate analysis: 2.820 × neoadjuvant − 0.033 × age + 1.594 × ASA + 1.019 × stoma − 2.002 = score.

In the above formula, the variables take the values of 0 or 1 depending on whether neoadjuvant chemoradiotherapy is planned: (1 = yes,0 = no)ASA > 1(1 = yes,0 = no) and stoma planned (1 = yes,0 = no). If the value obtained from evaluating the formula is negative (or zero), then the time to surgery is under 8 weeks. If positive, then the time from diagnosis to surgery that is greater than 8 weeks is predicted.

## 4. Discussion

Preoperative surgical oncology pathways must comply with regulatory guidelines which typically mandate prompt surgical intervention. Through employing patient-specific optimisation interventions, prehabilitation is aimed at reducing perioperative morbidity and promoting rapid recovery following major surgery. Although we have shown that time to surgery was not associated with long-term overall survival [9], presently, there is insufficient evidence to extend the preoperative period meaning prehabilitation must therefore be incorporated into existing pathways [7]. To address the current lack of any method to reliably predict the time from diagnosis to surgery, we employed artificial neural networks to investigate accurate prediction, and therefore, possible tailoring of our institution's future prehabilitation programme was possible.

Utilising a prospective mature cohort, this exploratory analysis using data that would be available at the time of diagnosis demonstrated a sensitivity of 99% for both patients who had surgery within eight or twelve weeks with an overall accuracy around 90%. Lift charts show the ANN was up to five times more likely to correctly identify patients over random selection.

In these patients, who did not receive formal prehabilitation interventions, higher ASA scores were the most important potentially modifiable factor identified suggesting prehabilitation could be of benefit to this group. ASA scores are only very rarely downgraded, but improvements in objective fitness measurements and functional tests whilst awaiting surgery have been shown [8, 17].

Our results were supported by traditional uni- and multivariate analyses as well as logistic regression modelling confirming the internal validity of this approach and suggesting ANN can be prospectively used to predict individual patient's time to surgery for colorectal cancer. ANN analysis was not seen to be informative for the four-week analysis suggesting a number of factors that were not included in the ANN model such as patient choice and local logistics which are important considerations.

As an example of machine learning, ANN hold a number of potential advantages over traditional biomedical statistical analyses [11, 13] which typically utilise multivariate regression tests when comparing the relationships of multiple variables. Although familiar to surgeons, these highly complex traditional statistical methods hold a number of drawbacks. Tests are applied retrospectively to cohort data, and importantly, the answer provided is typically a snapshot and only relevant for the studied timepoint. It cannot be assumed that the result is automatically applicable to future patients or other centres. The accuracy of ANN predictive models would be expected to improve as more data becomes available, and the network could also respond to evolutions in multidisciplinary care. The ANN can be regularly retrained and explored to establish which variables hold the highest clinical significance, allowing targeted quality improvement interventions. ANN may be more appropriate to report the nonlinear relationships between multiple variables and can be surprisingly simple to initiate. There is a large drive towards the application of machine learning to complex problem domains to identify the important factors and identify otherwise hidden relationships. Open source software such as TensorFlow™ (Google, CA, USA) has been released to facilitate the expansion of this strategy to new areas. Machine learning and ANN offer a number of possible uses in surgical practice, and several successful applications have been reported [14–16].

Although widely used by technology and analytical companies, ANN presents some considerations when applied to healthcare scenarios. Even for common clinical situations, the size of datasets is unlikely to match those analysed by industry and therefore may not be able to accommodate a large number of input variables or achieve the highest level of accuracy. To a degree, this could be offset by performing state-wide registry-based ANN testing subject to data protection and confidentiality considerations. This could inform benchmarking and quality assurance processes as well as informing public health and healthcare policy. A central limit theorem could also be expected to balance out many factors that can influence patient care. Matched institutional-specific ANN could help inform planning for individual patients as this network would incorporate local provider factors that are likely to influence time to surgery. Our findings now require a larger scale study and validation in external centres.

Although our pragmatic study is the first to use ANN in preoperative care through exploring time to surgery in a laparoscopic and enhanced recovery cohort, our findings should be interpreted in view of a number of limitations. Our ANN was based upon patient and tumour data given their influence on clinical decision-making processes but did not incorporate healthcare provider considerations or factors such as patient choice which will influence time to surgery. The 13-year study period is wide which risks these organisational factors impacting on time to surgery.

Given our results, the building of a more complex ANN is now justified. Increasing amounts of data should allow a larger number of endpoints such as weekly periods to be reported rather than four-week blocks that can be argued to hold less clinical relevance. The number of layers and connections within large ANN could result in overfitting of data causing significant associations that are due to chance rather than causal relationships with clinical significance. Our sample size prevented further division into training, validation, and testing groups which could have offset this issue. Therefore, all ANN models should be sufficiently explored before individual patient or wide-scale interventions or alterations in care for are considered. ASA is a crude measure of patient fitness. Future modelling would benefit from a more precise, individualised, and modifiable measure of patient fitness. As no further data on patient comorbidity or potential for improvement is known for this cohort, further opportunities for a more complex future ANN design exist. External validation of our findings is also required before wider application is considered.

## 5. Conclusion

Artificial neural networks using demographic and diagnosis data successfully predicted those likely to wait less than eight and twelve weeks for surgery. This finding could assist the personalisation of preoperative care including tailored prehabilitation programmes.

## Figures and Tables

**Table 1:** Patient and tumour demographics. The entire cohort was dichotomised using each studied timepoint. As expected, a number of significant differences are observed.

Time from diagnosis to curative laparoscopic surgery (*n*)	Whole cohort 668 (100%)	4 weeks or less 296 (44.3%)	Greater than 4 weeks 372 (55.7%)	*p*	8 weeks or less 537 (80.4%)	Greater than 8 weeks 131 (19.6%)	*p*	12 weeks or less 578 (86.5%)	Greater than 12 weeks 90 (13.5%)	*p*
Age (mean)	70	71	70	0.339	71	69	**0.009**	67	71	**0.001**
Males (*n*)	389 (58.2%)	169 (57.1%)	220 (59.1%)	0.594	300 (55.9%)	89 (67.9%)	**0.012**	325 (56.2%)	64 (71%)	**0.008**
Body mass index (median)	26	26	26	0.601	26	26	0.565	26	26	0.444
ASA (*n*)										
1	79 (11.8%)	47 (15.9%)	32 (8.6%)	0.07	72 (13.4%)	7 (5.3%)	**0.008**	74 (12.8%)	5 (5.6%)	0.167
2	420 (62.9%)	176 (59.5%)	244 (65.6%)		340 (63.3%)	80 (61.1%)		365 (63.1%)	55 (61.1%)	
3	161 (24.1%)	68 (23%)	93 (25%)		118 (22%)	43 (32.8%)		131 (22.3%)	30 (33.3%)	
4	4 (0.6%)	2(0.7%)	2 (0.5%)		3 (0.6%)	1 (0.8%)		3 (0.5%)	0	
Unknown	4 (0.6%)	3(1%)	1 (0.3%)		4 (0.7%)	0		5 (0.9%)	0	
Tumour location										
Colon	407 (60.9%)	217 (73.3%)	190 (51.1%)	**<0.001**	357 (66.5%)	50 (38.2%)	**<0.001**	382 (61.1%)	25 (27.8%)	**<0.001**
Rectum	261 (39.1%)	79 (26.7%)	182 (48.9%)		180 (33.5%)	81 (61.8%)		196 (33.9%)	65 (72.2%)	
Tumour stage (TNM 5th edition)										
0	42 (6.3%)	16 (5.4%)	26 (7%)	0.249	32 (6%)	10 (7.6%)	0.098	34 (5.9%)	8 (8.9%)	0.109
1	138 (20.7%)	49 (16.6%)	89 (23.9%)		109 (20.3%)	29 (22.1%)		117 (20.2%)	21 (23.3%)	
2	220 (32.9%)	115 (38.9%)	105 (28.2%)		189 (35.2%)	31 (23.7%)		200 (34.6%)	20 (22.2%)	
3	208 (31.1%)	91 (30.7%)	117 (31.5%)		165 (30.7%)	43 (32.8%)		179 (31%)	29 (32.2%)	
4	36 (5.4%)	16 (5.4%)	20 (5.4%)		25 (4.7%)	11 (8.4%)		28 (4.8%)	8 (8.9%)	
Unknown	24 (3.6%)	9 (3%)	15 (4%)		17 (3.2%)	7 (5.3%)		20 (3.5%)	4 (4.4%)	
Neoadjuvant treatment	57 (8.5%)	4 (1.4%)	53 (14.2%)	**<0.001**	14 (2.6%)	43 (32.8%)	**<0.001**	16 (2.8%)	41 (45.6%)	**<0.001**
Stoma planned	235 (35.1%)	72 (24.3%)	163 (43.8%)	**<0.001**	155 (28.9%)	80 (61.1%)	**<0.001**	169 (29.2%)	66 (73.3%)	**<0.001**

**Table 2:** The relative importance of each variable used in each ANN construction is displayed for each analysis. The requirement of neoadjuvant therapy understandably holds importance for 8 and 12 weeks. American Society of Anaesthesiologists score (ASA) is seen to hold importance at all timepoints.

Time from diagnosis to surgery	ANN variable	Importance	Normalised importance
4 weeks	Gender	.077	33.7%
	ASA	.153	67.5%
	Tumour location	.143	63.0%
	Stage	.157	69.4%
	Neoadjuvant treatment	.071	31.3%
	Stoma planned	.079	34.8%
	Age	.093	40.9%
	Body mass index	.227	100.0%

8 weeks	Gender	.050	18.9%
	ASA	.169	63.7%
	Tumour location	.007	2.6%
	Stage	.149	56.0%
	Neoadjuvant treatment	.266	100.0%
	Stoma planned	.062	23.4%
	Age	.173	65.0%
	Body mass index	.124	46.7%

12 weeks	Male gender	.034	10.9%
	ASA	.148	48.1%
	Tumour location	.027	8.9%
	Stage	.125	40.5%
	Neoadjuvant treatment	.308	100.0%
	Stoma planned	.076	24.7%
	Age	.234	76.1%
	Body mass index	.048	15.7%

**Table 3:** Predictive ANN accuracy for each analysis. Training consisted of 70% of the cohort selected and random with the remaining patients used for testing.

Time from diagnosis to surgery	Sample	Observed	Predicted		Percent correct
			Time to surgery under (*n*)	Time to surgery greater (*n*)	
4 weeks	Training	4 weeks or less	100	93	51.8%
		>4 weeks	69	166	70.6%
		Overall percent	39.5%	60.5%	62.1%
	Testing	4 weeks or less	43	44	49.4%
		>4 weeks	33	85	72.0%
		Overall percent	37.1%	62.9%	62.4%

8 weeks	Training	8 weeks or less	342	8	97.7%
		>8 weeks	63	28	30.8%
		Overall percent	91.8%	8.2%	83.9%
	Testing	8 weeks or less	158	2	98.8%
		>8 weeks	20	12	37.5%
		Overall percent	92.7%	7.3%	88.5%

12 weeks	Training	12 weeks or less	366	11	97.1%
		>12 weeks	29	30	50.8%
		Overall percent	90.6%	9.4%	90.8%
	Testing	12 weeks or less	168	2	98.8%
		>12 weeks	16	11	40.7%
		Overall percent	93.4%	6.6%	90.9%

**Table 4:** Multivariate analysis using data displaying odds ratio for surgery delayed over eight weeks.

8 weeks	*B*	Sig.	Exp (*B*) Odds ratio	95% CI for odds ratio	
				Lower	Upper
Age	-0.033-	.009	0.967	0.943	0.992
Neoadjuvant treatment	2.820	.000	16.769	8.179	34.381
Stoma planned	1.019	.001	2.771	1.543	4.976
ASA	1.594	.008	4.925	1.515	16.016
Constant	-2.002-	.033	.135		

ASA: American Society of Anaesthesiologists score.

## Data Availability

The data used within this study has not been made available to maintain patient confidentiality.

## Supplementary Materials

Supplementary Materials Supplementary Figure 1a-c: ROC gain and lift charts for the four-week ANN analysis. ANN modelling is shown to lack clinical utility for this timepoint. DtL: diagnosis to laparoscopy. Supplementary Figure 2: the neuronal links and strengths for the twelve-week ANN. Overall results are similar to the 8-week model although the relative importance of individual factors differs (Table 2). ANN behaviour and interaction between the layers are explained in Figure 1 legend.
