# Supplementary material for: Artificial Neural Network Individualised Prediction of Time to Colorectal Cancer Surgery
Source: Gastroenterol Res Pract. 2019 Jul 9;2019:1285931. doi: 10.1155/2019/1285931 (PMC6652036; doi:10.1155/2019/1285931)

Supplementary Description:

Supplementary Figure 1a-c ROC gain and lift charts for the four-week ANN analysis. ANN modelling is shown to lack clinical utility for this timepoint. DtL – Diagnosis to laparoscopy.

Supplementary Figure 2 The neuronal links and strengths for the twelve-week ANN. Overall results are similar to the 8-week model although the relative importance of individual factors differs (table 2). ANN behaviour and interaction between the layers is explained in the figure 1 legend.

Supplementary figure 1a-c

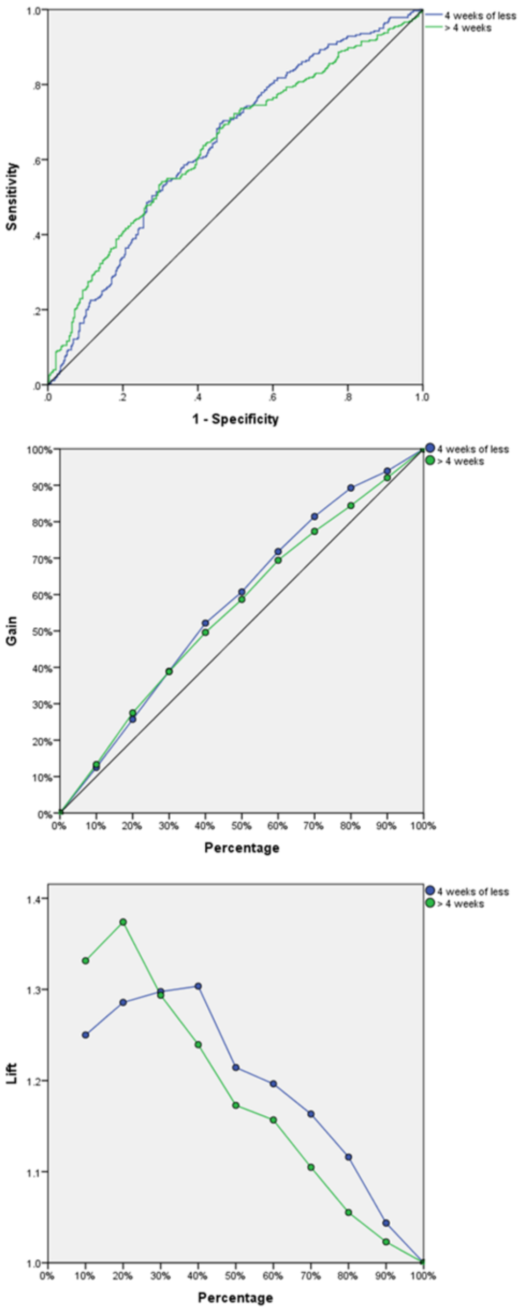

Supplementary figure 2

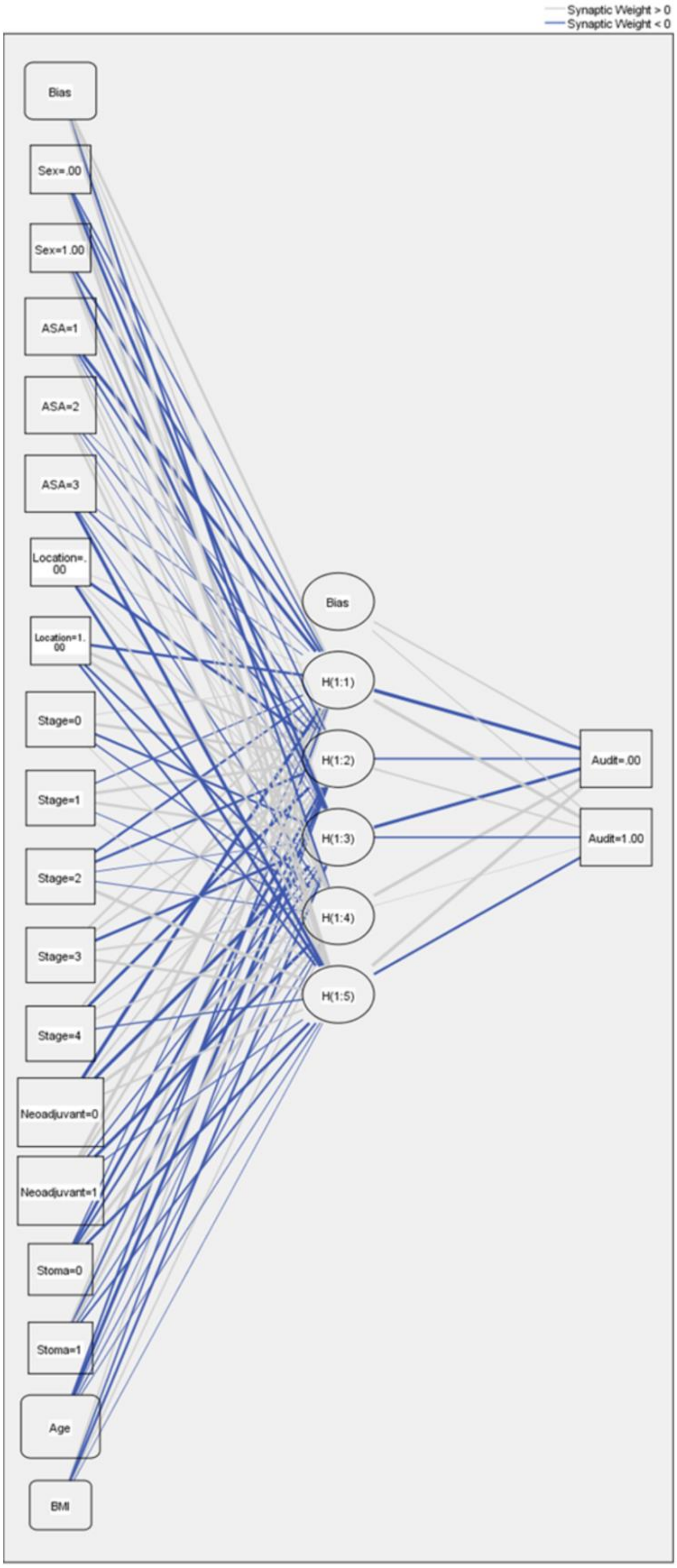

Supplement: Supplementary Materials — Supplementary Figure 1a-c: ROC gain and lift charts for the four-week ANN analysis. ANN modelling is shown to lack clinical utility for this timepoint. DtL: diagnosis to laparoscopy. Supplementary Figure 2: the neuronal links and strengths for the twelve-week ANN. Overall results are similar to the 8-week model although the relative importance of individual factors differs (Table 2). ANN behaviour and interaction between the layers are explained in Figure 1 legend. [file 1285931.f1.pdf]
